# Improvement on the Repair Effect of Electrochemical Chloride Extraction Using a Modified Electrode Configuration

**DOI:** 10.3390/ma11020225

**Published:** 2018-02-01

**Authors:** Wei Feng, Jinxia Xu, Linhua Jiang, Yingbin Song, Yalong Cao, Qiping Tan

**Affiliations:** College of Mechanics and Materials, Hohai University, Nanjing 210098, China; wei_feng@hhu.edu.cn (W.F.); jianglinhua@hhu.edu.cn (L.J.); babybin@hotmail.com (Y.S.); ctcuag@hhu.edu.cn (Y.C.); 171308060009@hhu.edu.cn (Q.T.)

**Keywords:** electrochemical chloride extraction, modified electrode configuration, electrochemical potential, corrosion current density, repassivation

## Abstract

To improve the repair effect of electrochemical chloride extraction, a modified electrode configuration is applied in this investigation. In this configuration, two auxiliary electrodes placed in the anodic and cathodic electrolytes were used as the anode and cathode, respectively. Besides this, the steel in the mortar was grounded to protect it from corrosion. By a comparative experiment, the potential evolution, various ions concentrations (Cl^−^, OH^−^, Na^+^, and K^+^) in different mortar depths, the corrosion potential, and the current density of the steel were measured. The results indicate that compared to electrochemical chloride extraction with the traditional electrode configuration, this electrochemical chloride extraction method with a modified electrode configuration has a similar chloride removal ratio. Besides this, potential of steel is just about 800 mV for a saturated calomel electrode (SCE) during the treatment, which did not reach the hydrogen evolution potential. The phenomenon of the accumulation of OH^−^, Na^+^, and K^+^ did not occur when the modified electrode configuration is applied. Additionally, higher corrosion potentials and lower corrosion current rates were measured after performing electrochemical chloride extraction with the modified electrode configuration. Additionally, it is a short period of time for the steel to go from activation to passivation. On this basis, the modified electrode configuration may overcome the drawbacks of electrochemical chloride extraction.

## 1. Introduction

The chloride-induced corrosion of steel is one of the main causes of a reduction in the durability of reinforced concrete structures exposed to a chloride-laden environment [1,2,3]. To prolong the service life of a chloride-contaminated concrete structure, the technique called “electrochemical chloride removal” or “electrochemical chloride extraction” has been extensively applied [4,5,6] due to its merits of high efficiency, relatively low cost, and having no need for maintenance. Electrochemical chloride extraction normally requires us to mount an anode at the concrete surface and have the steel reinforcement act as the cathode. A high current density (direct current (DC)) for a few weeks is applied. The applied current moves the chloride ions in the concrete away from the steel bar toward the anode, and thereby the chloride ions are removed from concrete [7]. Accordingly, an electrochemical chloride extraction treatment can remarkably reduce the chloride content near the reinforcement, leading to the reduction of the corrosion current density or the re-passivation of the steel, therefore significantly prolonging the service life of the chloride-contaminated concrete structure.

Despite this, there are still several defects in the electrochemical chloride extraction method reported in the literature. First, hydrogen gas evolves at the reinforcing steel because of the low cathodic potential that is induced by the high current density. It provides a warning that there is a risk of hydrogen-induced brittle fracture, especially for pre-stressed concrete [8]. Second, during the electrochemical chloride extraction, the reaction at the cathode can be described as follows:

2H_2_O + O_2_ + 4e^−^ → 4OH^−^ (1)

This reaction can increase the alkalinity near the surface of the steel, which is favorable for protecting the steel from corrosion. However, an alkaline attack on the steel will happen if the pH value is raised to 14 [9]. Third, cations such as Na^+^ and K^+^ in the pore solution of the concrete move simultaneously toward the steel cathode under the applied electric current, which induces the accumulation of sodium and potassium at the interface between the steel and concrete. This has been demonstrated to increase the risk of an alkali–silica reaction in concrete containing potentially reactive aggregates [10]. Besides this, the accumulation of sodium and potassium can reduce the bond strength between the steel and the concrete [11,12]. Finally, it has been found that the corrosion current of steel is significantly increased when the power supply is disconnected at the termination of the electrochemical chloride extraction [13,14]. As a result, the above defects have limited the wide application of electrochemical chloride extraction in real concrete structures.

According to the electrochemical mechanism of electrochemical chloride extraction, the above defects are mainly attributed to the direct connection of the steel with the cathode of the DC power supply. The reason is due to the fact that this connection induces a cathode reaction on the steel surface, resulting in hydrogen evolution and a significant increase of alkalinity. In addition, the electro-migration of sodium and potassium ions towards the cathode of the steel leads to the accumulation of sodium and potassium at the interface between the steel and the concrete. Based on this, it can be anticipated that if the steel reinforcement is not directly connected to the cathode of the DC power supply and used as the cathode during electrochemical chloride extraction, the drawbacks mentioned above may be defeated.

To improve the repair effect of electrochemical chloride extraction, a modified electrode configuration is applied to electrochemical chloride extraction in this study. In this configuration, two auxiliary electrodes positioned in anodic and cathodic electrolytes were used as the anode and cathode, respectively. Besides this, the steel in the mortar was grounded to guard against corrosion. By a comparative experiment, the potential evolution, the content of various ions (Cl^−^, OH^−^, Na^+^, and K^+^) in different mortar depths and electrolytes, the corrosion potential, and the corrosion current density of the steel were measured to evaluate the effectiveness of electrochemical chloride extraction with the modified electrode configuration.

## 2. Materials and Methods

### 2.1. Materials and Specimen Preparation

**Figure 1:**
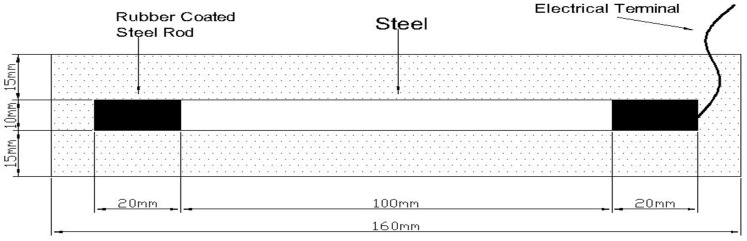
Detailed drawing of mortar specimens.

The cement applied in our work was No. 42.5 ordinary Portland cement made in China. Its oxide composition is indicated in Table 1. The fine aggregate was river sand with a fineness modulus of 2.65. A mortar specimen with a water/cement ratio of 0.55 and a sand/cement ratio of 2.5 was prepared. In order to simulate chloride-contaminated concrete, chloride ions (from calcium chloride) with content in 2.0% by mass of cement were added into the mortar specimen. Besides this, the specimen had a size of 40 mm × 40 mm × 160 mm with a centrally embedded steel bar (10 mm in diameter). The surface of the steel was cleaned physically prior to the application. The detailed drawing of the mortar specimens is presented in Figure 1.

Moreover, after one day of casting in plastic moulds, all of the paste samples were demolded and then cured in a 95% humidity chamber at 20 ± 2 °C for 28 days.

### 2.2. Electrochemical Treatment

**Figure 2:**
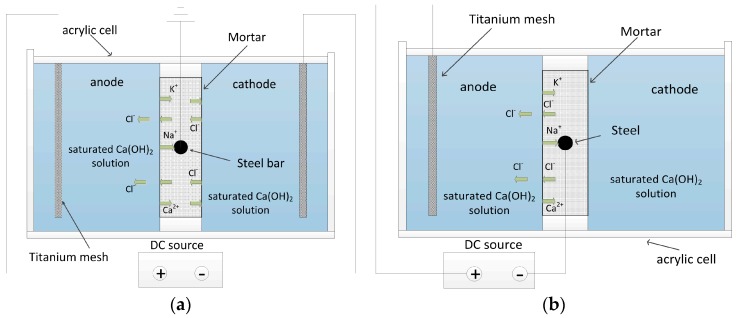
Detailed experimental configurations for the modified electrode configuration (MEC) treatment (**a**) and the traditional electrode configuration (TEC) treatment (**b**). DC: direct current.

A rectangular electrolytic tank (24 cm × 16 cm × 6 cm) made from acrylic sheets was separated into two identical compartments by placing the as-fabricated mortar specimen in the middle of the tank. For the aim of conducting electrochemical chloride extraction with the modified electrode configuration (abbreviated as MEC treatment), a saturated calcium hydroxide solution was readied for use as cathodic and anodic electrolytes. Platinized titanium meshes were applied as the anode and cathode. In addition, the steel in the mortar specimen was grounded to be protected against corrosion. The detailed experimental configuration for the MEC treatment is indicated in Figure 2a. In contrast, the steel in the mortar specimen was directly connected to the cathode of the DC power supply during electrochemical chloride extraction with the traditional electrode configuration (abbreviated as TEC treatment). The mortar specimen was placed into the saturated calcium hydroxide solution. The detailed experimental configuration for the TEC treatment is indicated in Figure 2b.

The MEC and TEC treatment periods for each electrical field applied were 28 days. A 100-watt DC power supply was used. For the MEC treatment, the positive and negative poles of the power supply were connected with the titanium mesh in the anode and the cathode, respectively. As a comparison, during the TEC treatment, the positive and negative poles of the power supply were connected with titanium mesh and steel, respectively.

Three different constant current densities (2.0, 4.0, and 8.0 A/m^2^, based on the mortar specimen surface) were used. The saturated calcium hydroxide solution was replaced every two days to maintain a stable composition. All of the MEC and TEC treatments were performed at ambient temperature.

### 2.3. Measurements

#### 2.3.1. Steel Potential Measurement

The electrochemical potential of the steel was monitored periodically during the TEC treatment and the MEC treatment. The saturated calomel reference electrode as the reference electrode was positioned on the surface of the mortar. Then, the electrochemical potential was measured with a recorder connected to a computer.

#### 2.3.2. Cl^−^, OH^−^, Na^+^, and K^+^ Contents

During the TEC treatment and the MEC treatment, the anodic electrolytic solutions were collected every two days for determining the cumulative amounts of chloride extraction with time. Besides this, the treated specimens were cut into slices from the mortar surface to the steel. Each slice was cut with a 5 mm thickness and powdered until it passed through a sieve of 0.16 mm. Then, 20 g powders were added into 200 mL distilled waters and 200 mL nitric acid solutions so as to determine the free Cl^−^ content (the number of Cl^−^ ions remaining free in the pore solution of the mortar) and the total Cl^−^ content (including free chloride and the chloride ions bound in the cement hydrates), respectively. The mixtures were vigorously stirred for a period, and then left to stand for 24 h. All of the chloride contents were measured by potentiometric titration with 0.01 M silver nitrate (AgNO_3_).

Moreover, the suspensions applied to determine the free chloride content were also employed to measure the OH^−^, Na^+^, and K^+^ contents. Prior to the measurement, the suspensions were filtered. The OH^−^ content of the leachate was measured using a pH-meter. Besides this, the contents of sodium (Na^+^) and potassium (K^+^) ions in the leachates were determined by flame atomic absorption spectrometry.

#### 2.3.3. Corrosion Potential and Corrosion Current Density of the Steel

As soon as the TEC treatment and the MEC treatment were finished, the mortar specimens were transferred to an atmospheric environment (20 °C and 60% relative humidity (RH)). The corrosion potential and corrosion current density for the steel reinforcement in the mortar specimen after the TEC treatment and the MEC treatment were continuously monitored for three months by the linear polarization method. For the linear polarization measurement, the Partstat 2273 advanced potentiostat/Galvanostat/frequency response analyser system (AMETEK Corporation, Berwyn, PA, USA) was applied. Besides this, the saturated calomel electrode (SCE) and a platinum electrode had been connected to work as the reference electrode and the auxiliary electrode, respectively. The linear polarization scan was carried out between −10 mV and +10 mV with respect to the rest potential (E_corr_). The potential was applied at a rate of 0.1 mV/s. The polarization resistance (R_p_) obtained by the linear polarization measurement was introduced into the Stern–Geary equation to calculate the corrosion current density (I_corr_). The Stern–Geary equation is:

I_corr_/B = R_p_ (2)

where B is a constant, which is assumed to be 26 mV (its value has been determined by means of calibration against mass loss measurements) [15,16].

## 3. Results

### 3.1. Change of Steel Potential with Time

**Figure 3:**
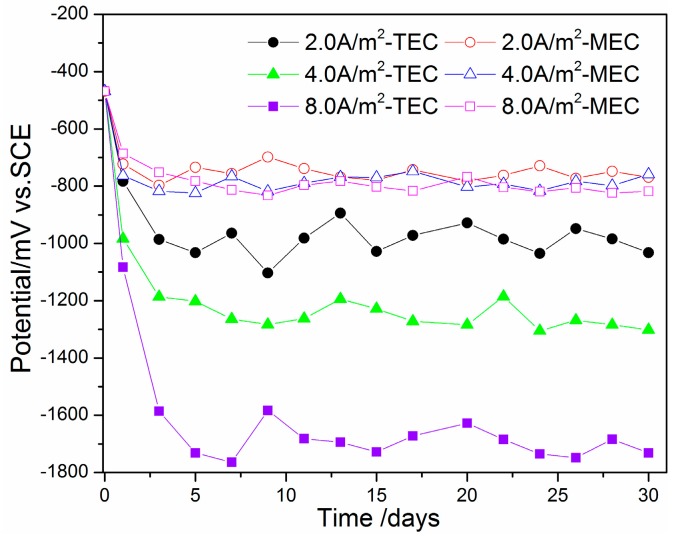
Change of steel potential with time during the TEC treatment and the MEC treatment. SCE: saturated calomel electrode.

Figure 3 shows the change of steel potential with time in the mortars during the TEC treatment and the MEC treatment. Prior to the treatments, the potential of the steel was about −500 mV versus the SCE. When the TEC treatment was applied, the potentials of the steel are sharply decreased. The ultimate value of the potential is maintained at −1000 mV versus the SCE for the specimen with a 2.0 A/m^2^ current density. With the increase of applied current density, the absolute value of the steel’s potential is obviously increased. The lowest value of the steel’s potential for the TEC treatment is about −1800 mV versus the SCE. Compared to the TEC treatment, the potential of the steel for the MEC treatment is obviously higher. The ultimate value of the steel’s potential is only −800 mV versus the SCE. In addition, the potential of the steel does not obviously change with the increase of applied current density during the MEC treatment.

### 3.2. Extracted Chloride, Free Chloride, and Total Chloride Contents

**Figure 4:**
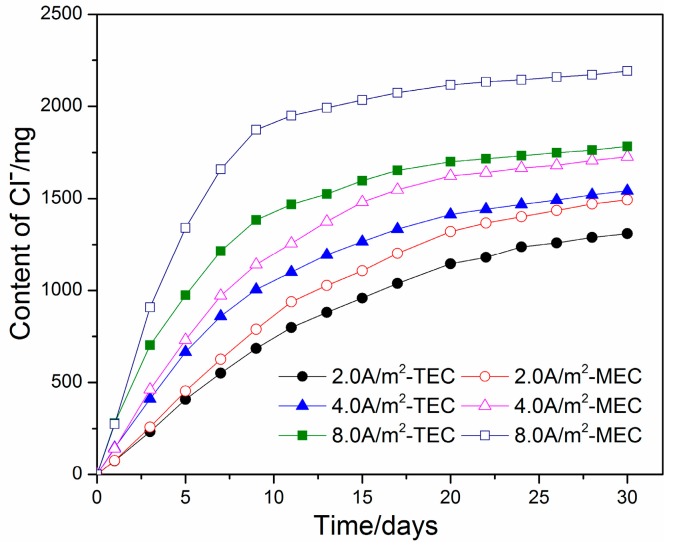
Cumulative amounts of chloride in the anolyte during the TEC treatment and the MEC treatment.

**Figure 5:**
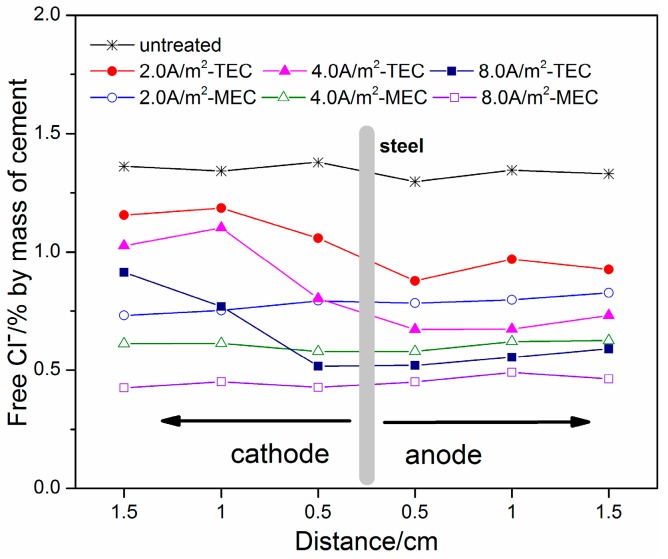
Free chloride concentrations versus locations from the steel to the anolyte or the catholyte (untreated, after the TEC treatment and the MEC treatment).

**Figure 6:**
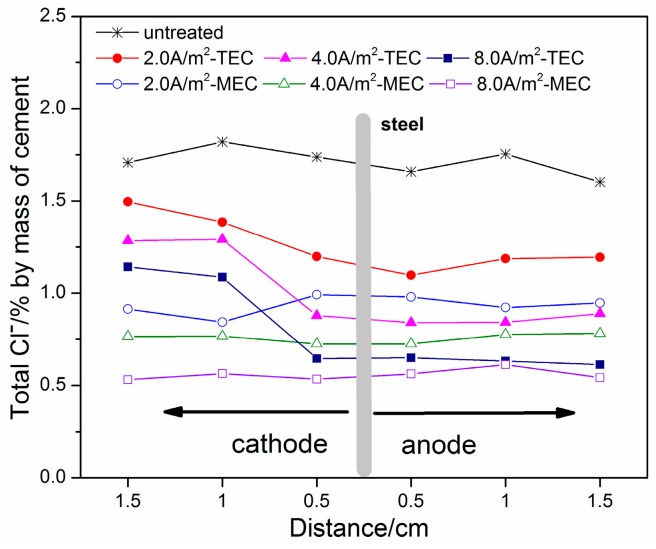
Total chloride concentrations versus locations from the steel to the anolyte or the catholyte (untreated, after the TEC treatment and the MEC treatment).

Variations in the extracted chloride content in the anolyte versus time for the TEC treatment and the MEC treatment are presented in Figure 4. For both the TEC treatment and the MEC treatment, most of the chloride contents are extracted during the first 2 weeks of application. As the time of treatment is further increased, the extracted chloride content in the anolyte is slowly increased. Compared to the TEC treatment, the chloride content in the anolyte for the MEC treatment is slightly higher when the same current density is applied. The results show that the MEC treatment did not reduce, but increased the chloride removal ratio. A similar result can be also concluded from Figure 5 and Figure 6.

Figure 5 and Figure 6 show the profiles of free chloride and total chloride in the mortar specimens after the TEC treatment and the MEC treatment, respectively. Besides this, the profiles of free chloride and total chloride for the untreated mortar specimens are indicated in Figure 5 and Figure 6 in order to make a comparison. It can be seen in Figure 5 that the free chloride contents before the TEC treatment and the MEC treatment have a uniform distribution in the mortar specimens. The average value is about 1.4% (by mass of cement). Besides this, a similar result can be obtained for the distribution of the free chloride content after the MEC treatment. However, the free chloride content is obviously decreased, indicating the obvious chloride removal capacity of this treatment. Furthermore, with the increase of applied current density, the free chloride content is further reduced. Compared to the MEC treatment, the free chloride contents in the specimens after the TEC treatment display a non-uniform distribution. The chloride content in the depth close to the external anode is relatively lower. However, higher chloride content in the opposite direction of the external anode can be found. With the increase of applied current density, the trends for the non-uniform distribution of free chloride content are more remarkable. Furthermore, a higher chloride removal ratio for the MEC treatment in the same depth is obtained compared to that for the TEC treatment. Compared to the profiles of free chloride, the profiles of total chloride exhibit a similar regularity (see Figure 6). Compared to the TEC treatment, the total chloride content for the MEC treatment is relatively lower under the same applied current density, indicating a slightly better chloride removal capacity.

### 3.3. OH^−^, Na^+^, and K^+^ Contents

**Figure 7:**
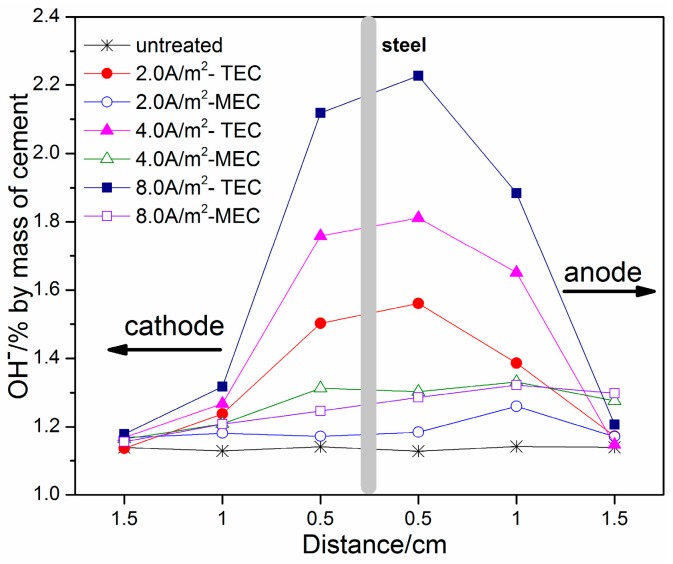
Concentrations of OH^−^ ions as a function of location (untreated, after the TEC treatment and the MEC treatment).

Figure 7 presents the OH^−^ contents in relation to the locations for the TEC treatment and the MEC treatment. From the figure, an increase in applied current density leads to an increase in OH^−^ content at the same position for the TEC treatment. The content of OH^−^ ions near the steel surface is increased by 30% (by mass of cement) when a current density of 2.0 A/m^2^ is applied. However, this value is increased up to 80% by mass of cement when a current density of 8.0 A/m^2^ is applied. Compared to the TEC treatment, the content of OH^−^ near the steel surface is remarkably reduced for the MEC treatment, only having the value of 1.2~1.3% (by mass of cement). Besides this, with the increase of the current density, the content of OH^−^ is slightly increased. Furthermore, the content of OH^−^ near the steel surface in the mortar after the MEC treatment is only slightly higher than that in the mortar without the treatment.

**Figure 8:**
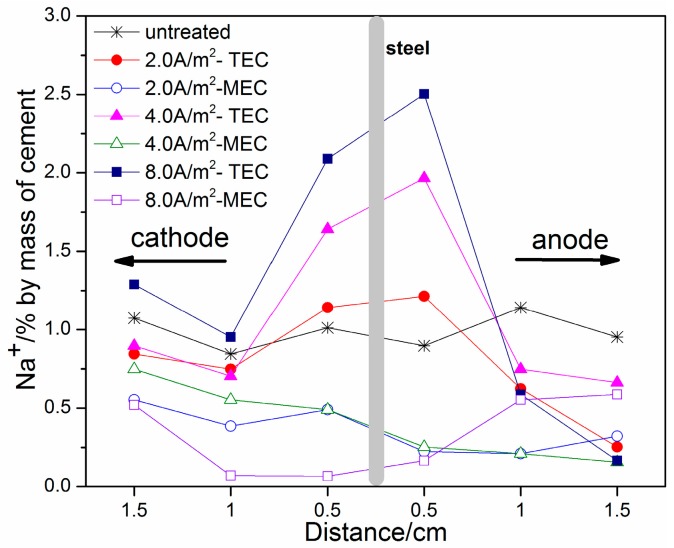
Concentrations of Na^+^ ions as a function of location (untreated, after the TEC treatment and the MEC treatment).

**Figure 9:**
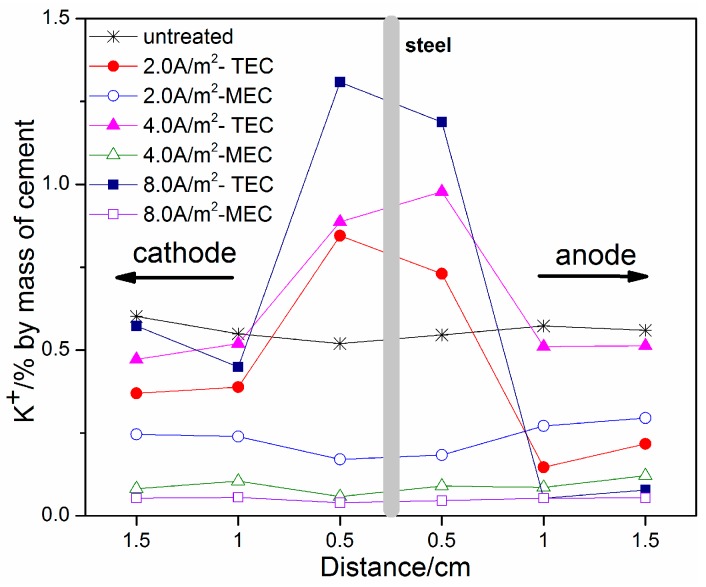
Concentrations of K^+^ ions as a function of location (untreated, after the TEC treatment and the MEC treatment).

Figure 8 and Figure 9 show the profiles of Na^+^ and K^+^ contents, respectively. It can be seen that both Na^+^ and K^+^ contents exhibit a similar regularity of change with location after the TEC treatment. An obvious increase of Na^+^ and K^+^ contents near the steel surface can be obtained, which is similar to the previous literature [8,12]. With the increase of applied current density, the Na^+^ and K^+^ contents are further increased. In contrast, the Na^+^ and K^+^ contents near the steel surface are not increased, but are softly reduced after the MEC treatment. Therefore, it can be concluded that the MEC treatment cannot induce the accumulation of sodium and potassium near the steel surface.

### 3.4. Corrosion Potential and Corrosion Current Density of the Steel

**Figure 10:**
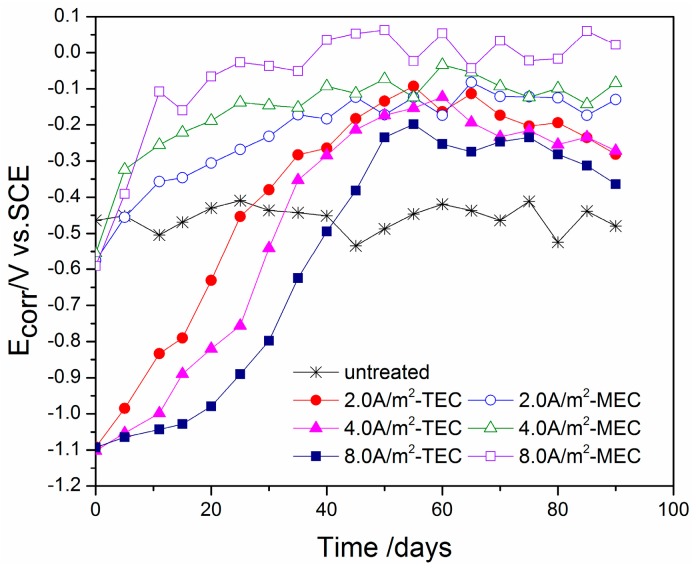
Changes of steel corrosion potential with time (untreated, after the TEC treatment and the MEC treatment).

Figure 10 presents the changes of measured corrosion potentials with time after the TEC treatment and the MEC treatment. The corrosion potential for the specimen without the treatment is only about −500 mV versus the SCE. However, the corrosion potentials for all of the TEC treatments have a lower initial value of about −1100 mV versus the SCE. It is sharply increased with the increase of time. After a time of 60 days, the corrosion potentials for the TEC treatments attain a stable value. In contrast, the corrosion potentials for all of the MEC treatments have the value of −600 mV versus the SCE, which is slightly lower than those for the specimen without the treatment. Furthermore, it only takes 40 days for all of the MEC treatments to attain a stable value of corrosion potential. We can also discover that the E_corr_ values of the TEC treatment specimens are all lower than those of the MEC treatment when the value has achieved stability.

**Figure 11:**
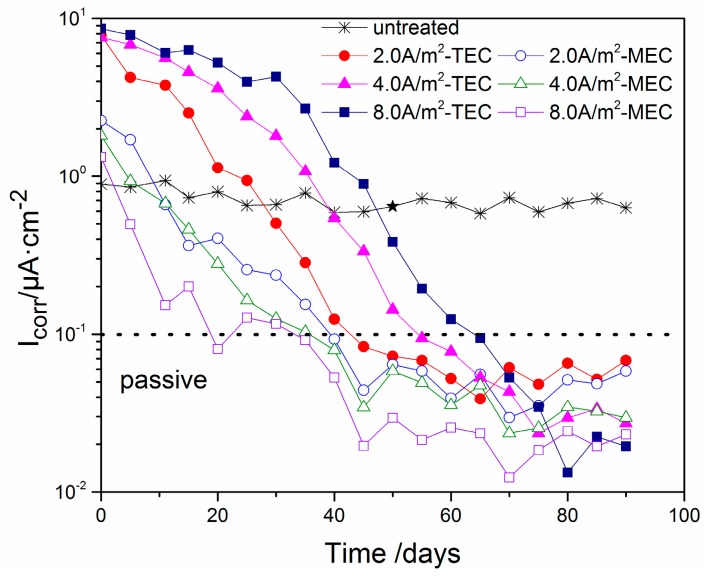
Changes of the steel corrosion current density with time (untreated, after the TEC treatment and the MEC treatment).

The changes of measured corrosion current density with time after the TEC treatment and the MEC treatment are indicated in Figure 11. As can be seen, the corrosion current density of the specimen without the treatment has a value of about 0.9 µA/cm^2^. However, the initial values of the corrosion current density are increased to about 10 µA/cm^2^ after the TEC treatments. Besides this, with the increase of time, it is obviously decreased. Similar to the corrosion potential, the corrosion densities for all of the TEC treatments attain a stable value after the waiting time of 60 days. Compared with this, the initial values of the corrosion current density are only more than 1.3 µA/cm^2^ after the MEC treatments, which are only slightly more than those for the specimen without the treatment. Furthermore, it only takes 40 days for all of the MEC treatments to attain a stable value of corrosion current density.

## 4. Discussion

In the TEC treatment, the steel reinforcement is directly connected to the cathode of the power supply. Under this circumstance, a high negative potential of the steel can be attained due to the application of a high current density. Also, the cathode reaction (1) normally happens, producing a large amount of OH^−^ so that the alkalinity of the pore solution near the steel surface is remarkably increased. Besides this, the cations such as Na^+^ and K^+^ in the pore solution of the concrete move simultaneously toward the cathode of the steel, which induces the accumulation of sodium and potassium at the interface between the steel and concrete. In contrast, the MEC treatment has an obvious difference in the experimental setup with the TEC treatment. Two auxiliary electrodes are installed on both sides of the mortar. As a result, the potential of the steel is expected to be increased, and the chemical reaction (1) does not take place on the steel surface. Besides this, the accumulation of sodium and potassium does not happen at the interface between the steel and the concrete. Furthermore, the steel reinforcement is grounded in the MEC treatment. Such an arrangement is helpful for protecting steel from corrosion by the leakage of current, and increases obviously the potential of the steel.

From the obtained results, the negative potentials of the steel in the mortars ranges from −1000 mV to approximately 1800 mV versus the SCE during the TEC treatment, which is dependent on the applied current density. According to the literature [17,18], such extremely low potentials can lead to hydrogen evolution near the steel surface, which can increase the expansion stress around the steel. Once the expansion stress attains a critical value, a micro-crack in the mortar around the steel will happen. Besides this, the hydrogen evolution may cause the loss of steel elasticity so that a brittle fracture of the steel will happen [19]. Accordingly, it is generally assumed that the TEC treatment cannot be applicable to pre-stressed concrete structures [20,21].

Compared to the TEC treatment, the potentials of the steel for the MEC treatment are obviously increased. The values for all of the MEC treatments with different current densities are only maintained at around −800 mV versus the SCE. According to the literature [17], the electrochemical reaction that takes place on the steel surface is dependent on the potential of the steel. The lower the potential of the steel is, the more easily hydrogen evolution happens. Moreover, the hydrogen evolution can be affected by some other factors, such as the alkalinity of the pore solution and the saturation of the concrete on the steel surface. As a rule, a potential of steel less than −1200 mV versus the SCE will induce hydrogen evolution. Contrary to this, a potential of steel more than −1100 mV versus the SCE will not lead to hydrogen evolution, but a reaction of dissolved oxygen [17,18]. This reaction cannot produce hydrogen evolution so that hydrogen embrittlement of the steel can be avoided by the MEC treatment.

The capacity for chloride removal of the MEC treatment is slightly better than that of the TEC treatment. Beyond this, the experimental results show the uneven distribution of free and total (Figure 6) chloride in the mortar specimens after the TEC treatment. A higher content of free chloride at a location far from the anolyte can diffuse towards the steel, which may cause corrosion again [18]. Based on physical considerations, this behavior can be attributed to the fact that the electrical field does not pass through the part of mortar far away from the anolyte. In contrast, the free and total chloride contents in the mortar specimens after the MEC treatment are uniformly distributed. It can be explained by the current with a uniform distribution throughout the entire mortar specimen during the MEC treatment. Consequently, a chloride ion in any part of the mortar can be removed effectively and uniformly. As a result, the MEC treatment may reduce the risk of secondary corrosion due to the redistribution of surviving chloride ions in the mortar.

The large amounts of OH^−^ produced at the steel surface after the TEC treatment are mainly due to the cathode reaction. Although the increase of OH^−^ content near the steel surface is normally assumed to be helpful for protecting steel from corrosion, too high a pH value will accelerate corrosion. According to the literature [9], a pH value that is too high will put the steel in the alkaline corrosion region of the E/pH diagram (the equilibrium phases of iron in aqueous solutions as a function of pH and electrochemical potential vs. SHE (Standard Hydrogen Electrode)). In addition, as described previously in the introduction, the high alkalinity on the steel surface after the TEC treatment increases the risk of an alkali–silica reaction. Therefore, it is necessary to maintain a moderate alkalinity to protect the steel from corrosion and guard against the alkali–silica reaction. In contrast, the MEC treatment only can increase slightly the alkalinity near the steel surface due to the electro-migration of the hydroxyl ions in the electrolytic solution to the steel surface in the mortar. Such a small increase of the alkalinity not only is helpful to protect the steel from corrosion, but will not lead to the alkaline corrosion of the steel. Also, it cannot increase obviously the risk of an alkali–silica reaction.

In the TEC treatment, the Na^+^ and K^+^ concentrated at the interface will react with OH^−^ ions produced by the cathode reaction so as to form hydroxide. Then, the further reaction of alkaline hydroxide with calcium silicate (C-S-H) can lead to the softening of the concrete. This result has been assumed to be the main cause for a strength decrease of the bonding between the steel reinforcement and the concrete [19,22]. In contrast, the MEC treatment does not induce a strength decrease of the bonding due to not forming accumulations of sodium and potassium at the interface between the steel and the concrete.

Some previous works [9,14,23] have indicated that the extraction treatment has positive effects on the corrosion potential (E_corr_) of the steel bars. In this study, the corrosion potential of the steel in the mortar specimens is also moved to the positive direction after the TEC treatment and the MEC treatment, indicating that both the TEC treatment and the MEC treatment reduce the possibility of corrosion. However, the change trends of the potential are obviously different between the TEC treatment and the MEC treatment when the power supply is disconnected. The corrosion potential of the steel after the TEC treatment is maintained at an extremely low level for a long duration due to the slow restoration of the steel’s polarization. In contrast to this, the corrosion potential of steel after the MEC treatment only has a moderate value, and it is easy to attain a stable value because the steel reinforcement is grounded to be depolarized.

Similar to the potential of steel, similar change trends for the corrosion density can be obtained after the TEC treatment and the MEC treatment. Furthermore, it is well-known that the corrosion current density of 0.1 µA/cm^2^ has been extensively accepted as the critical value for the determination of the reinforcement state from active to passive [24,25]. By means of this standard, the corrosion state of the steel reinforcement after the TEC treatment and the MEC treatment can be determined. Compared to the TEC treatment, the re-passivation of the steel after the MEC treatment can happen more quickly. Besides this, the corrosion current density at the equilibrium is relatively lower.

From the obtained results, the MEC treatment has a slightly higher chloride removal ratio than the TEC treatment. Besides this, the MEC treatment may defeat the defects of the traditional electrochemical chloride extraction technology, such as: hydrogen embrittlement, alkaline corrosion, and alkali–silica reaction. Also, the steel reinforcement after the MEC treatment may be easier to obtain re-passivation for than after the TEC treatment. Therefore, the MEC treatment shows promise for application in real concrete structures on a wider range, even for pre-stressed reinforced concrete.

## 5. Conclusions

To improve the repair effect of electrochemical chloride extraction, a modified electrode configuration is presented in this study. By a comparative experiment, the potential evolution, the content of various ions (Cl^−^, OH^−^, Na^+^, and K^+^) in different mortar depths and electrolytes, the corrosion potential, and the corrosion current density of steel were measured. Some conclusions have been obtained:

(1) The potential of the steel during the MEC treatment has a value of −800 mV versus the SCE, which is far lower than that during the TEC treatment. Besides, the applied current density does not change obviously the potential of the steel during the MEC treatment. This potential cannot induce hydrogen evolution so that hydrogen embrittlement of the steel can be avoided by using the MEC treatment.
(2) For both the TEC treatment and MEC treatment, most of the chloride contents are extracted during the first 2 weeks of application. Compared to the TEC treatment, the chloride content in the anolyte and the free chloride content in the mortar are obviously decreased, but the total chloride content in the mortar for the MEC treatment is increased when the same current density is applied. Therefore, the MEC treatment has a better chloride removal ratio than the TEC treatment.
(3) An obvious increase of OH^−^, Na^+^, and K^+^ contents near the steel surface can be obtained for the TEC treatment. In contrast, the OH^−^, Na^+^, and K^+^ contents near the steel surface are softly reduced after the MEC treatment. Therefore, the MEC treatment cannot induce the accumulation of OH^−^, Na^+^, and K^+^ near the steel surface.
(4) The corrosion potentials for the specimens after all of the TEC treatments have an extremely lower initial value than the corrosion potential of the specimen without the treatment. Contrary to this, all of the potentials of the steel have a higher value when the equilibrium state of the steel is attained. However, the corrosion potentials for the specimens after all of the MEC treatments are slightly lower than the corrosion potential for the specimen without the treatment. The initial values of the corrosion current density after the MEC treatments are lower than those after the TEC treatments. In addition, the re-passivation of the steel is more quickly attained after the MEC treatment.

## Figures and Tables

**Table 1:** Oxide composition of cement used (% by mass).

Oxide	CaO	SiO_2_	Al_2_O_3_	Fe_2_O_3_	MgO	K_2_O	Na_2_O	Total Cl	SO_3_	Ignition Loss
Composition (wt.%)	57.27	24.99	9.32	3.11	0.86	0.83	0.28	0.05	1.13	2.16
